# A burrowing annelid from the early Cambrian

**DOI:** 10.1098/rsbl.2024.0357

**Published:** 2024-10-09

**Authors:** Xiaoyu Yang, M. Teresa Aguado, Jie Yang, Christoph Bleidorn

**Affiliations:** ^1^ Yunnan Key Laboratory for Palaeobiology, Institute of Palaeontology, Yunnan University, Kunming 650500, People’s Republic of China; ^2^ Animal Evolution and Biodiversity, University of Göttingen, Göttingen 37073, Germany

**Keywords:** Annelida, cephalic cage, burrowing, early Cambrian, Xiaoshiba biota

## Abstract

Soft-bodied fossils of annelids from the Cambrian are relatively rare but provide vital insights into the early evolution and diversification of annelids. Here we describe a new annelid, *Xiaoshibachaeta biodiversa* gen. et sp. nov., from the early Cambrian (Stage 3) Xiaoshiba biota of Kunming, Yunnan Provence, China. This worm is obliquely oriented in the sediment, and is characteristic of a cephalic cage-like structure formed by the anteriorly directed parapodia and long chaetae of chaetiger 1, strongly suggesting an endobenthic lifestyle. This first report of an annelid worm from the Xiaoshiba biota provides the earliest known plausible evidence of burrowing behaviour in Annelida. Phylogenetic analyses recover *X. biodiversa* in the polytomy with other crown-group Annelida, indicating that the evolution of cephalic cage in Annelida is most likely convergent.

## 1. Introduction

Annelids are (mostly) segmented worms that inhabit a broad range of ecological niches in marine, freshwater and terrestrial environments. They exhibit diverse forms and lifestyles, ranging from metre-sized giants like *Eunice aphroditois* to millimetre-scale interstitial species such as Protodriliformia, and from burrowing to holopelagic habits [1]. Their soft-bodied nature makes their fossilization relatively rare [2] and often only hard structures of jaws (if present) are found in the fossil record [3]. Fortunately, exceptionally preserved fossil deposits such as the middle Cambrian Burgess Shale in Canada [4,5] and the Carboniferous Mazon Creek biota of Illinois [6,7] have yielded some remarkable whole-body fossils of annelids. These well-preserved specimens offer crucial insights into the morphology (e.g. head appendages, parapodia and chaetae), anatomical structures (e.g. musculature and nervous system) and lifestyles of ancient annelids [8]. In the past decade, an increasing number of whole-body annelid fossils from the early Cambrian have been unearthed, particularly in China [9–13]. Two of these newly discovered species, tubicolous *Dannychaeta* [11] and pelagic *Gaoloufangchaeta* [13] have been recovered by phylogenetic analyses within modern annelid clades, namely Palaeoannelida and Phyllodocida, respectively, indicating an early origin of the crown-group Annelida, and together with descriptions of other annelids, reveal that a great diversity of morphology and lifestyle of Annelida has been evolved during the ‘Cambrian Explosion’.

Here we describe a new annelid, *Xiaoshibachaeta biodiversa* gen. et sp. nov., from the early Cambrian Xiaoshiba Lagerstätte (Cambrian Series 2, Stage 3) of Kunming, South China. With its distinguishable cephalic cage formed by the anteriorly directed long chaetae of chaetiger 1, this new taxon represents the first annelid known from the Xiaoshiba Lagerstätte, and sheds new light on the early evolution as well as diversity in morphology and ecology of annelids.

## 2. Material and methods

### (a) Material

The specimen studied was collected from the early Cambrian (local Canglangpuan Stage) Xiaoshiba Lagerstätte, Kunming, Yunnan Province, southwestern China, and deposited at the Yunnan Key Laboratory for Palaeobiology (YKLP), Institute of Palaeontology, Yunnan University. The specimen number of the fossil is YKLP 12466.

### (b) Fossil imaging

Specimen was prepared manually with a needle to remove the matrix concealing the fossil under a LEICA M125 stereomicroscope. Photographs were taken using a KEYENCE VHX-6000, a KEYENCE VHX-7000 and a Leica DFC7000 T monochrome digital camera attached to a Leica M205 FA fluorescence stereomicroscope, and processed in Adobe Photoshop CS6 and CorelDRAW SE. Back scattered electron (BSE) image capture and energy dispersive X-ray analysis (EDS) were performed by using a JEOL JSM-IT500 SEM under low vacuum.

### (c) Phylogenetic analysis

Phylogenetic analyses are based on the published morphological matrix of Guo *et al*. [14]. Most taxa belonging to Brachiopoda and Mollusca and their related characters were removed. Two spioniform polychaetes with cephalic cages (Poecilochaetidae and Uncispionidae), fossil annelid *Aledochaeta* and new fossil *Xiaoshibachaeta* are added, thus resulting in a matrix with 91 taxa and 238 characters (no autopomorphies are included; electronic supplementary material). The annelid affinity of *Iotuba* from the Chengjiang biota [15] is controversial, and therefore has not been included here (see electronic supplementary material for detailed explanations). Maximum parsimony (MP) analysis was conducted using TNT v. 1.6 [16] under a traditional search with 1000 replicates. Implied weight (*k* = 10) was used to explore the effect of different degrees of homoplasy penalization to test the robustness of the dataset. Bayesian analyses with topological constraints were conducted using MrBayes 3.2.7a following the published MrBayes block of [14], with Mki model, gamma distribution for relative rates [17], and default priors for all parameters. A hundred million generations with 25% burnin were generated, and the analysis was stopped once the average deviation of split frequencies fell below 0.01. Convergence was assessed by an estimated sample size bigger than 200 and potential scale reduction factor equal to 1.00 for each parameter. Topological constraints contained are based on phylogenomic studies and current systematics of annelids [18,19] (electronic supplementary material).

## 3. Systematic palaeontology

Phylum Annelida Lamarck, 1809 [20].

*Xiaoshibachaeta biodiversa* gen. et sp. nov.

LSID. urn:lsid:zoobank.org:act:2DC02BE7-51F0-42D2-8BE3-26934C78DB96.

### (a) Etymology

*Xiaoshiba* refers to the section where the fossil was collected; *chaeta* (Latin), bristle, a typical characteristic of polychaetes; *biodiversa*, for biodiversity, to support all goals and targets of the Kunming–Montreal Global Biodiversity Framework.

### (b) Holotype

**Figure 1.**
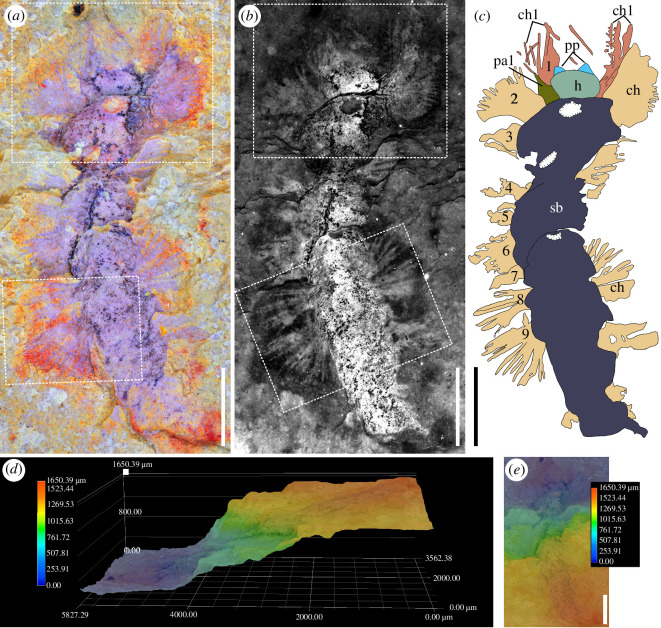
*Xiaoshibachaeta biodiversa* gen. et sp. nov. from the Xiaoshiba Lagerstätte, holoptype YKLP 12466, part. (*a*) Light microscopic image of the specimen, anterior is up. (*b*) Fluorescence image of the specimen. (*c*) Interpretive line drawing of the specimen; numbering denotes the parapodia. (*d,e*) Three-dimensional information revealed by KEYENCE microscopy, showing the worm penetrating obliquely into sediment. Abbreviations: ch, chaetae; ch1, first chaetae; h, head; pa1, first parapodia; pp, palps; sb, segmental body. Scale bars: 1 mm.

**Figure 2.**
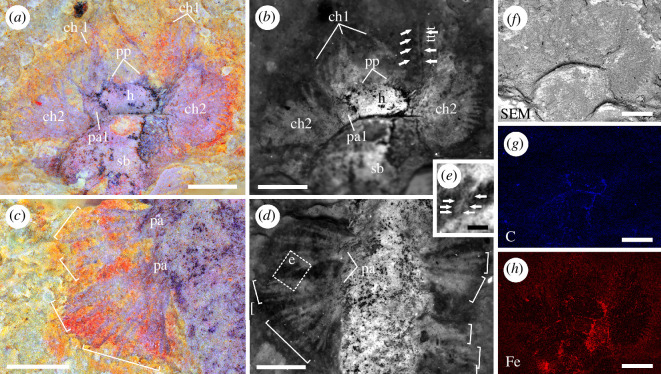
Detail of holoptype YKLP 12466, part. (*a*) Detail of anterior part of the specimen. (*b*) Anterior part of the specimen showing the elongate chaetae with side small spines (arrowed) of first chaetiger. (*c*) Well-preserved short parapodia with two bundles of capillary chaetae. (*d*) Detailed morphology of biramous parapodia and capillary chaetae. (*e*) Close-up of boxed area in (*d*), showing the detail of one chaeta with fine lateral spines (arrowed). (*f–h*) SEM-EDS images of the anterior part of the specimen. (*f*) Backscatter image. (*g*) Elemental mapping for carbon. (*h*) Elemental mapping for iron. (*a,c*) Close-ups of boxed areas in figure 1*a*. (*b,d*) Close-ups of boxed areas in figure 1*b*. Abbreviations: ch1, first chaetae; ch2, second chaetae; h, head; pa, parapodia; pa1, first parapodia; pp, palps; sb, segmental body. Scale bars: (*a–d, f–h*) 500 µm; (*e*) 100 µm.

YKLP 12466, part and counterpart (figures 1 and 2; electronic supplementary material, figures S2 and S3).

### (c) Occurrence

Lower part of the Hongjingshao Formation (Cambrian Series 2, Stage 3); Xiaoshiba Section, Kunming, Yunnan Provence, Southwest China.

### (d) Diagnosis

Annelid with elongated body. Prominent head elliptical, bearing a pair of short triangular palps. The trunk tapering posteriorly, possessing more than 14 chaetigers. Chaetiger 1 with large parapodia directed anteriorly, bearing elongate chaetae forming a cephalic cage-like structure. Subsequent chaetigers with biramous parapodia, each short and with long fan-like capillary chaetae. Approximately 4−8 chaetae on each notopodium and neuropodium.

### (e) Description

The worm is bilaterally symmetrical and dorsoventrally flattened (figure 1). The specimen is preserved as penetrating the micro-layers of the mudstone at a depth of about 1.65 mm (figure 1*d,e*). It is incomplete, lacking the posterior part, and has a preserved length of about 7 mm (excluding head appendages) (figure 1; electronic electronic supplementary material, figure S3). No characteristics indicating the dorsal and ventral sides of the specimen, such as eyes and mouth, have been preserved.

The head is differentiated from the trunk, and is elliptical in shape (*ca* 330 µm high and 570 µm wide) (figures 1*a*–*c* and 2*a,b*). A pair of short triangular structures situated at the anterior margin of the head (figures 1a–*c* and 2*a,b*) are interpreted as palp-like head appendages (see electronic supplementary material for an alternative interpretation of this anteriormost part of the specimen).

The trunk (segmented body) is elongated, and is wider than the head (figure 1). It bears at least 14 similar segments tapering gradually posteriorly (figure 1; electronic supplementary material, figure S3). The maximum width of segments is about 1.3 mm (figure 1*a*–*c*). A lobe projecting anteriorly from the anteriormost part of the trunk on one side has a broad base (*ca* 190 µm in width) and tapers apically (*ca* 370 µm in length) (figure 2*a,b*). It is interpreted as the parapodium of chaetiger 1. The first parapodium on the other side cannot be observed possibly due to the burrowing nature of the worm. Some slender strip-like structures associated with the first parapodia and having a length of about 0.8−1 mm and a width of about 13−37 µm (figure 2*a,b*) are here interpreted as chaetae (see electronic supplementary material for an alternative interpretation). Several chaetae can be observed with fine lateral spines (figure 2*b*).

The well-preserved parapodia of the subsequent chaetigers are short, and bear two bundles of laterally projected chaetae; each bundle has around 4−8 chaetae (figure 2*c,d*). Chaetae are simple and capillary, *ca* 540−790 µm in length and *ca* 40−75 µm in width (figure 2*c,d*). One chaeta is observed to have tightly set spines (*ca* 35 µm long) in the distal part (figure 2*e*). There is no evidence of aciculae or parapodial cirri.

Fluorescence imaging of the specimen allows the chaetae (weak fluorescence) to be distinguished from the relatively brighter head and trunk (figures 1*b* and 2*b*,*d*). The dark tissues on the head and trunk were identified by EDS as carbonaceous remains and iron oxides (figure 2*g*,*h*; electronic supplementary material, figure S1).

### (f) Remarks

In general morphology, *Xiaoshibachaeta* is similar to other Cambrian annelids (e.g. *Pygocirrus* [21]) in having elongated vermiform body and biramous parapodia with capillary chaetae. However, *Xiaoshibachaeta* differs from other Cambrian annelids by having an elliptical head and anteriorly directed parapodia with long capillary chaetae of chaetiger 1. *Xiaoshibachaeta* resembles *Dannychaeta* and *Gaoloufangchaeta* in having a differentiated head, but differs in its shape and head appendages [11,13]. The head of *Xiaoshibachaeta* is elliptical and has paired short palps while those of *Dannychaeta* and *Gaoloufangchaeta* are spade-shaped and with paired elongated palps, nearly round and paired antennae, palps and tentacular cirri, respectively [11,13].

## 4. Discussion

### (a) Morphology

The head of *Xiaoshibachaeta* is differentiated from the trunk as in *Dannychaeta* and *Gaoloufangchaeta* both of which are recovered by phylogenetic analyses within crown-group Annelida [11,13], suggesting that *Xiaoshibachaeta* probably is a part of the annelid crown group as well.

Chaetae of the first parapodia in *Xiaoshibachaeta* are longer than those of the remaining parapodia, and project anteriorly around the head, recalling the cephalic cage which is a typical characteristic of some sedentary annelids such as *Poecilochaetus* [22], *Trochochaeta* [23], Uncispionidae [24] and Flabelligeridae [25] (electronic supplementary material, figure S4). The former three taxa are small groups of Spioniformia and their cephalic cages are formed by the long capillary chaetae of chaetiger 1 and sometimes chaetiger 2; the latter one is a group of polychaetes called bristle-cage worms and the number of chaetigers involved in forming the cephalic cage range from one to six [19]. Some chaetae of *Xiaoshibachaeta* exhibit small spines laterally, showing a certain similarity to the plumose or spinose chaetae of *Poecilochaetus* [26]. *Xiaoshibachaeta* is also similar to these modern polychaetes in having paired head appendages and biramous parapodia without cirri. However, the head appendages of *Xiaoshibachaeta* are short and are located at the anterior margin of the head, indicating that they may have a prostomial origin, differing from those in the spioniforms and flabelligerids, which are long grooved and peristomial [22–25].

### (b) Palaeoecology

Part of the specimen is slightly S-shaped, and the chaetae on both sides of the same segment are in opposite directions (electronic supplementary material, figure S2a,b). This is similar to certain extant polychaetes in locomotion [19], indicating a possible undulatory movement for *Xiaoshibachaeta*.

A burrowing lifestyle is suggested for *Xiaoshibachaeta* based on both taphonomic and morphological considerations. The specimen of *Xiaoshibachaeta* is preserved to extend obliquely through the surrounding substrate. Such a penetrative state has also been described in some Cambrian endobenthic palaeoscolecids as evidence of their possible burrowing activity or escaping movement when being burial [27,28]. The anterior parapodia and elongate chaetae of *Xiaoshibachaeta* are comparable with those forming the cephalic cage of certain burrowing annelids such as *Poecilochaetus* which use the anterior-directed parapodia and cephalic chaetae of the first segment to excavate into the sediment [22]. There is no evidence indicating the feeding strategy of *Xiaoshibachaeta*. Unlike the long palps of *Poecilochaetus* and Flabelligeridae which function as a deposit and suspension feeding organ [22,25], the short palps of *Xiaoshibachaeta* are most likely sensory.

To date, there have been 19 species of soft-bodied annelids (including sipunculans) formally described from the Cambrian (electronic supplementary material, table S1), and they show great diversity in morphology and lifestyle. Most annelid worms were interpreted as epibenthic forms crawling over the bottom of the sea [29–31], some living in tubes they build or occupy [11,32], and one as pelagic [13]. Some annelids from the Burgess Shale such as *Peronochaeta* were assumed to be burrowers based on only indirect evidence (external morphologies) [4]. The finding of *Xiaoshibachaeta* represents the first plausible record of burrowing lifestyle in the Cambrian annelids.

### (c) Phylogenetic affinities

**Figure 3.**
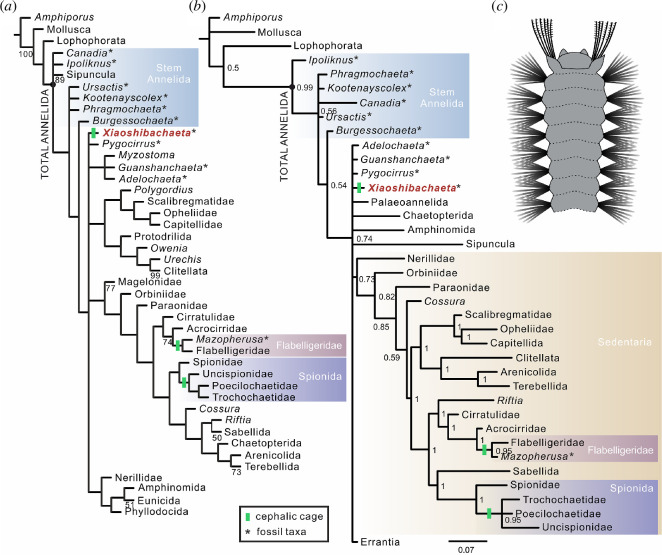
Phylogeny and reconstruction of *Xiaoshibachaeta*. (*a*) Simplified strict consensus tree of maximum parsimony under implied weight (*k* = 10, 16 most-parsimonious trees (MPTs), 874 steps, consistency index (CI) = 0.295, retention index (RI) = 0.647). Bootstrap support values (>50) are displayed. (*b*) Majority rule consensus tree of Bayesian inference. Numbers at nodes are posterior probabilities. Scale bar represents the expected changes per site. (*c*) Reconstruction of the anterior part of *Xiaoshibachaeta*.

Our phylogenetic results obtained using MP and Bayesian inference (BI) (figure 3; electronic supplementary material, figures S5 and S6) are consistent with those of previous analyses [11,13,14]: most of the Cambrian annelids are recovered as stem-group annelids; *Pygocirrus* fall into a polytomy with the crown-group annelids; the Cambrian *Gaoloufangchaeta*, as well as *Plumulites* and other Carbonaceous fossil taxa are placed within Errantia. MP and BI analyses are congruent in the phylogenetic position of *Xiaoshibachaeta*, placing this new taxon in the polytomy with other crown-group Annelida (figure 3; electronic supplementary material, figures S4 and S5). The cephalic cage has evolved in three groups which are not very closely related, including *Xiaoshibachaetae*, Spioniformia (Trochochaetidae, Poecilochaetidae and Uncispionidae) and Flabelligeridae. Convergent evolution of cephalic cages in these three taxa is the most parsimonious interpretation. The presence of a cephalic cage has also been reported for the enigmatic fossil taxon *Iotuba* [15]. However, as already mentioned, it is very doubtful that this fossil represents an annelid at all [13]. Moreover, the described arrangement of ‘spines’ around a retractile head, which do not affiliate with any visible parapodia-like structure, does not resemble similar structures found in any known annelid, thereby not supporting them as homologous.

## Data Availability

The data are provided as electronic supplementary material, which is available online [33].

## References

[1] Aguado MT, Capa M, Oceguera-Figueroa A, Rouse GW. 2014 Annelida. In The tree of life: evolution and classification of living organisms (eds P Vargas, R Zardoya), pp. 254–269. Sunderland, MA: Sinauer.

[2] Briggs DEG, Kear AJ. 1993 Decay and preservation of polychaetes: taphonomic thresholds in soft-bodied organisms. Paleobiology **19**, 107–135. (10.1017/S0094837300012343)

[3] Schwab KW. 1966 Microstructure of some fossil and recent scolecodonts. J. Paleontol. **40**, 416–423. (10.2307/1301671)

[4] Conway Morris S. 1979 Middle Cambrian polychaetes from the Burgess Shale of British Columbia. Phil. Trans. R. Soc. B **285**, 227–274. (10.1098/rstb.1979.0006)

[5] Nanglu K, Caron JB, Gaines RR. 2020 The Burgess Shale paleocommunity with new insights from Marble Canyon, British Columbia. Paleobiology **46**, 58–81. (10.1017/pab.2019.42)

[6] Thompson I. 1979 Errant polychaetes (Annelida) from the Pennsylvanian Essex fauna of northern Illinois. Palaeontogr. A **163**, 169–199.

[7] Shabica CW, Hay A. 1997 Richardson’s guide to the fossil fauna of Mazon Creek. Chicago, IL: Northeastern Illinois University.

[8] Parry LA, Tanner A, Vinther J. 2014 The origin of annelids. Palaeontology **57**, 1091–1103. (10.1111/pala.12129)

[9] Liu JN, Ou Q, Han J, Li JS, Wu YC, Jiao GX, He TJ. 2015 Lower Cambrian polychaete from China sheds light on early annelid evolution. Sci. Nat. **102**, 34. (10.1007/s00114-015-1285-4) PMC4446521 26017277

[10] Han J, Conway Morris S, Hoyal Cuthill JF, Shu DG. 2019 Sclerite-bearing annelids from the lower Cambrian of South China. Sci. Rep. **9**, 4955. (10.1038/s41598-019-40841-x) 30894583 PMC6426949

[11] Chen H, Parry LA, Vinther J, Zhai DY, Hou XG, Ma XY. 2020 A Cambrian crown annelid reconciles phylogenomics and the fossil record. Nature **583**, 249–252. (10.1038/s41586-020-2384-8) 32528177

[12] Zhao J, Li YJ, Selden PA. 2023 A new primitive polychaete with eyes from the lower Cambrian Guanshan biota of Yunnan Province, China. Front. Ecol. Evol. **11**, 1128070. (10.3389/fevo.2023.1128070)

[13] Yang X, Aguado MT, Helm C, Zhang Z, Bleidorn C. 2024 New fossil of Gaoloufangchaeta advances the origin of Errantia (Annelida) to the early Cambrian. R. Soc. Open Sci. **11**, 231580. (10.1098/rsos.231580) 38601033 PMC11004674

[14] Guo J *et al*. 2022 A Cambrian tommotiid preserving soft tissues reveals the metameric ancestry of lophophorates. Curr. Biol. **32**, 4769–4778.(10.1016/j.cub.2022.09.011) 36170853

[15] Zhang ZF, Smith MR, Ren XY. 2023 The Cambrian cirratuliform Iotuba denotes an early annelid radiation. Proc. R. Soc. B **290**, 20222014. (10.1098/rspb.2022.2014) PMC9890102 36722078

[16] Goloboff PA, Morales ME. 2023 TNT version 1.6, with a graphical interface for MacOS and Linux, including new routines in parallel. Cladistics **39**, 144–153. (10.1111/cla.12524) 36682054

[17] Lewis PO. 2001 A likelihood approach to estimating phylogeny from discrete morphological character data. Syst. Biol. **50**, 913–925. (10.1080/106351501753462876) 12116640

[18] Struck T. 2019 Phylogeny. In Handbook of zoology. Annelida. Volume 1: Annelida basal groups and Pleistoannelida, Sedentaria I (eds G Purschke, M Böggemann, W Westheide), pp. 37–68. Berlin, Germany: De Gruyter. (10.1515/9783110291582-002)

[19] Rouse G, Pleijel F, Tilic E. 2022 Annelida. Oxford, UK: Oxford University Press. (10.1093/oso/9780199692309.001.0001)

[20] Lamarck J. 1809 Philosophie zoologique. Paris, France: Dentu.

[21] Vinther J, Eibye-Jacobsen D, Harper DAT. 2011 An early Cambrian stem polychaete with pygidial cirri. Biol. Lett. **7**, 929–932. (10.1098/rsbl.2011.0592) 21733871 PMC3210688

[22] Blake JA, Maciolek NJ. 2020 7.4.2. Poecilochaetidae Hannerz, 1956. In Handbook of zoology. Annelida. Volume 2: Annelida basal groups and Pleistoannelida, Sedentaria II (eds G Purschke, M Böggemann, W Westheide), pp. 103–119. Berlin, Germany: De Gruyter. (10.1515/9783110291681-003)

[23] Blake JA, Maciolek NJ. 2020 7.4.3. Trochochaetidae Pettibone, 1963. In Handbook of zoology. Annelida. Volume 2: Annelida basal groups and Pleistoannelida, Sedentaria II (G Purschke, M Böggemann, W Westheide), pp. 120–135. Berlin, Germany: De Gruyter.

[24] Blake JA, Maciolek NJ. 2020 7.4.4. Uncispionidae Green, 1982. In Handbook of zoology. Annelida. Volume 2: Annelida basal groups and Pleistoannelida, Sedentaria II (eds G Purschke, M Böggemann, W Westheide), pp. 136–144. Berlin, Germany: De Gruyter. (10.1515/9783110291681-004)

[25] Salazar-Vallejo SI. 2019 7.3.1.6. Flabelligeridae Saint-Joseph, 1894. In Handbook of zoology. Annelida. Volume 1: Annelida basal groups and Pleistoannelida, Sedentaria I (eds G Purschke, M Böggemann, W Westheide), pp. 398–421. Berlin, Germany: De Gruyter.

[26] Magalhães WF, Bailey-Brock JH, Santos CSG. 2015 A new species and two new records of Poecilochaetus (Polychaeta: Poecilochaetidae) from Hawaii. J. Mar. Biol. Assoc. UK **95**, 91–100. (10.1017/S002531541400109X)

[27] Huang DY, Chen JY, Zhu MY, Zhao FC. 2014 The burrow dwelling behavior and locomotion of palaeoscolecidian worms: new fossil evidence from the Cambrian Chengjiang fauna. Palaeogeogr. Palaeoclimatol. Palaeoecol. **398**, 154–164. (10.1016/j.palaeo.2013.11.004)

[28] Yang Y, Zhang X, Zhao Y, Qi Y, Cui L. 2018 New paleoscolecid worms from the early Cambrian north margin of the Yangtze Platform, South China. J. Paleontol. **92**, 49–58. (10.1017/jpa.2017.50)

[29] Nanglu K, Caron JB. 2018 A new Burgess Shale polychaete and the origin of the annelid head revisited. Curr. Biol. **28**, 319–326.(10.1016/j.cub.2017.12.019) 29374441

[30] Parry LA, Caron JB. 2019 Canadia spinosa and the early evolution of the annelid nervous system. Sci. Adv. **5**, eaax5858. (10.1126/sciadv.aax5858) 31535028 PMC6739095

[31] Osawa H, Caron JB, Gaines RR. 2023 First record of growth patterns in a Cambrian annelid. R. Soc. Open Sci. **10**, 221400. (10.1098/rsos.221400) 37122950 PMC10130728

[32] Nanglu K, Caron JB. 2021 Symbiosis in the Cambrian: enteropneust tubes from the Burgess Shale co-inhabited by commensal polychaetes. Proc. R. Soc. B **288**, 20210061. (10.1098/rspb.2021.0061) PMC8150028 34034516

[33] Yang X, Aguado MT, Yang J, Bleidorn C. 2024 Supplementary material from: A burrowing annelid from the early Cambrian. Figshare. (10.6084/m9.figshare.c.7477897)39378985

